# Recent Advances in Zein-Based Nanocarriers for Precise Cancer Therapy

**DOI:** 10.3390/pharmaceutics15071820

**Published:** 2023-06-26

**Authors:** Wenquan Huang, Fei Yao, Shuangyan Tian, Mohao Liu, Guijin Liu, Yanbin Jiang

**Affiliations:** 1 College of Medicine and Health Science, China Three Gorges University, Yichang 443002, China; huangwenquan@ctgu.edu.cn (W.H.); yaofei@ctgu.edu.cn (F.Y.); tianshuangyan@ctgu.edu.cn (S.T.); 202110550021015@ctgu.edu.cn (M.L.); 2 School of Pharmaceutical Sciences, Hainan University, Haikou 570100, China; 3 School of Chemistry and Chemical Engineering, South China University of Technology, Guangzhou 510640, China

**Keywords:** zein, nanocarrier, anticancer, precision medicine

## Abstract

Cancer has emerged as a leading cause of death worldwide. However, the pursuit of precise cancer therapy and high-efficiency delivery of antitumor drugs remains an enormous obstacle. The major challenge is the lack of a smart drug delivery system with the advantages of biodegradability, biocompatibility, stability, targeting and response release. Zein, a plant-based protein, possesses a unique self-assembly ability to encapsulate anticancer drugs directly or indirectly. Using zein as a nanotherapeutic pharmaceutic preparation can protect anticancer drugs from harsh environments, such as sunlight, stomach acid and pepsin. Moreover, the surface functionalization of zein is easily realized, which can endow it with targeting and stimulus-responsive release capacity. Hence, zein is an ideal nanocarrier for the precise delivery of anticancer drugs. Combined with our previous research experiences, we attempt to review the current state of the preparation of zein-based nanocarriers for anticancer drug delivery. The challenges, solutions and development trends of zein-based nanocarriers for precise cancer therapy are discussed. This review will provide a guideline for precise cancer therapy in the future.

## 1. Introduction

Cancer mortality and morbidity are increasing rapidly worldwide, posing a serious threat to human health [1]. Over the past few decades, researchers have developed many new cancer drugs, such as cisplatin, carboplatin, platinum oxalate, paclitaxel (PTX), gefitinib, erlotinib and afatinib. However, most of these cancer drugs are not approved by the Food and Drug Administration (FDA) due to their poor water solubility, low bioavailability and weak targeting ability, which lead to low efficacy and various toxic side effects [2,3,4]. For example, platinoid drugs have the characteristics of a broad spectrum and strong efficacy against cancer, but they often cause nephrotoxicity, gastrointestinal reactions, ototoxicity, marrow toxicity, etc. [5,6]. Therefore, improving the efficacy and reducing the side effects are the keys to the development of anticancer drugs.

Recently, various advanced materials have provided new opportunities for the delivery of anticancer drugs [7,8,9]. Advanced materials such as micelles, liposomes, animal protein, plant protein, metal and non-metallic nanoparticles (NPs) have obvious advantages in improving drug stability, solubility, targeting ability and drug sustained release [9,10,11,12]. Based on these materials, researchers have designed a variety of advanced multifunctional drug delivery systems (DDSs), which provide tremendous advances in anticancer drug delivery [13,14]. However, traditional DDSs are confronted with two major problems with regard to precise delivery. On the one hand, DDSs inevitably cause immune stimulation or immunosuppressive effects. Traditional DDSs have failed to achieve the desired therapeutic effect in vivo due to multiple drug delivery obstacles such as immune system clearance, complex tumor microenvironment and plasma protein adsorption, leading to the shielding of targeted peptides [15,16,17]. On the other hand, the enhanced permeability and retention (EPR) effect has been used in traditional DDSs to enhance solid tumor target ability [18]. Hassan et al. [19] reported that zein NPs modified with polyethylene glycol (PEG) had a longer half-life and higher drug retention rate (80.42 ± 1.16%), which ensured the drug could penetrate hepatocellular carcinoma by EPR effect. However, this effect has been questioned by researchers. Pozo et al. [20] reported that nanoparticles with a size bigger than 100–150 nm could not achieve tumors’ passive targeting by EPR effect. Nichols et al. [21] reported that EPR did not necessarily realize targeted aggregation and sometimes drug carriers did not exhibit a higher therapeutic effect compared with free drug systems in clinical trials. Additionally, Caro et al. [22] reported that the presence of the blood–brain tumor barrier prevents the EPR effect of metallic nanoparticles. Therefore, other methods should be developed. Although researchers have developed various active carriers over the past decades, there has been no breakthrough in targeting DDSs, and few nanotechnology-based drug preparations have been successfully marketed. Therefore, designing nanodrug carriers to realize efficient utilization of anticancer drugs has become an important goal for pharmaceutical researchers.

**Figure 1:**
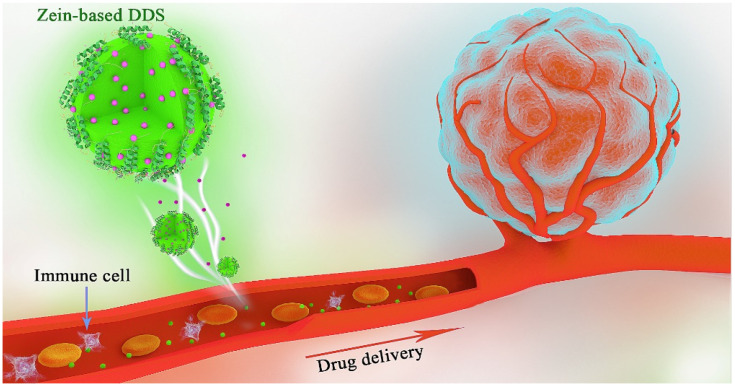
The rationale behind using zein-based nanocarriers for cancer therapy.

Zein is a plant-based protein with various advantages including low cytotoxicity, abundant sources, high drug binding capacity and biocompatibility [23,24]. The unique protein structure of zein provides the potential for a variety of ligand modifications to achieve the stability and specificity of zein-based DDSs [9,25]. The rationale behind using zein-based nanocarriers for cancer therapy is shown in Figure 1. After injection or oral administration, zein-based DDSs encapsulated with anticancer drugs enter the blood circulation. To avoid clearance by the immune cells, the DDS can be modified with PEG or cancer cell membrane, which exhibit a long circulation half-life [7,26]. To obtain the targeting ability, zein-based DDSs can be conjugated with the targeting polypeptide or tumor microenvironment response substances, which will increase the concentration of anticancer drugs in the tumor tissue [9,27]. Then, precise cancer therapy can be achieved. For oral administration, zein-based DDSs can protect unstable anticancer drugs from degradation by enzymes or strongly acidic environments; as such, they show a long residence time within the gastrointestinal tract [28].

**Figure 2:**
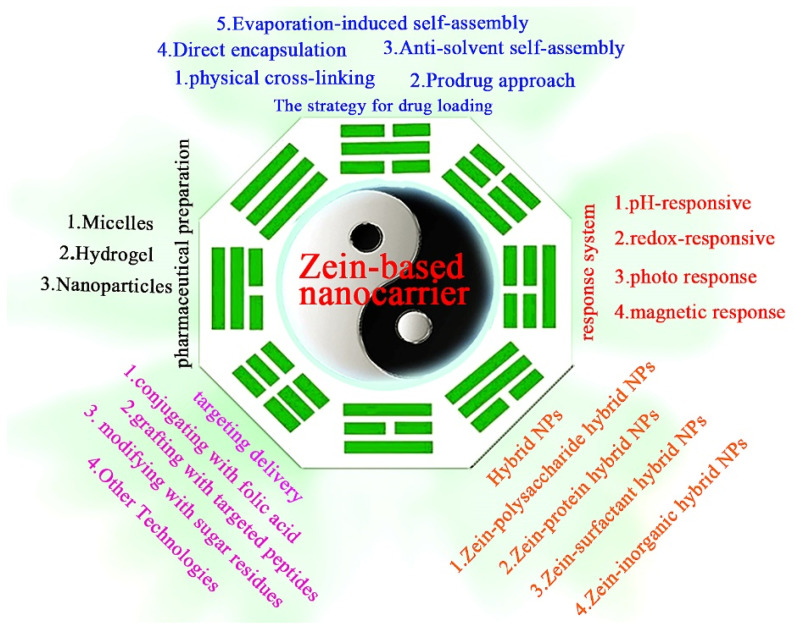
The current role of zein-based nanocarriers for precise cancer therapy.

Zein can be modified with NPs such as gold, metal–organic frameworks and mesoporous silica to broaden its applications [9,17,29]. However, currently commercially available zein is a mixture, and it is difficult to judge where zein molecules are modified. The preparation of zein to form DDSs is a complex self-assembly and co-assembly process. The strategy for drug loading is the key point in order to further apply zein in the delivery of anticancer drugs. In this review, the status of zein for anticancer drug delivery is summarized (Figure 2), which provides deeper insights into precise cancer therapy of zein-based carriers.

## 2. Preparation of Zein-Based Nanocarriers for Anticancer Drug Delivery

### 2.1. The Strategy for Drug Loading

The current strategies for the preparation of zein nano-DDS include physical cross-linking, evaporation-induced self-assembly, anti-solvent self-assembly, microfluidic technology, chemical synthesis of the prodrug and direct encapsulation. The different strategies for drug loading result in different particle nanostructures, encapsulation efficiency, drug loading, drug delivery behavior (rapid release or controlled release) and cell uptake. Hence, the different drug loading strategies result in different therapeutic effects.

#### 2.1.1. Physical Cross-Linking

**Figure 3:**
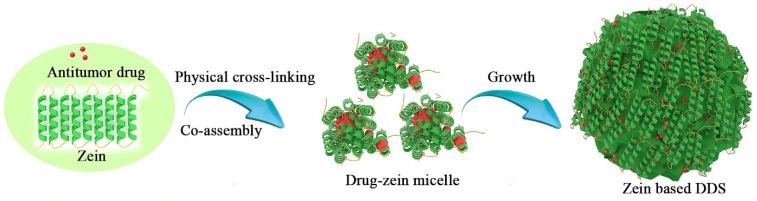
The loading drug process of zein by physical cross-linking.

Physical interactions between polymer chains provide a platform for the encapsulation of various types of bioactive compounds [30]. As shown in Figure 3, hydrophobic zein is an ideal carrier for the loading of lipophilic anticancer drugs based on physical cross-linked methods (PCL), where anticancer drugs can interact with zein through hydrophobic, electrostatic interactions, hydrogen bonding and van der Waals forces. When the solvent environment is changed, the co-assembly between zein and anticancer drugs occurs, and the drugs are encapsulated in the zein micelles by the physical interactions. Then, these micelles grow into zein-based DDSs. Seok et al. [31] used zein to encapsulate curcumin molecules by electrostatic interaction and hydrogen bonding, where hyaluronic acid (HA) acted as a stabilizer and targeting agent. The obtained HA–zein–curcumin nanogels could effectively inhibit cancer cell growth both in vitro and in vivo. Elzoghby et al. [32] developed shell-crosslinked zein nanocapsules for oral codelivery of exemestane and resveratrol. The obtained NPs possessed an enhanced cytotoxicity for MCF-7 and 4T1 breast cancer cells.

#### 2.1.2. Evaporation-Induced Self-Assembly

**Figure 4:**
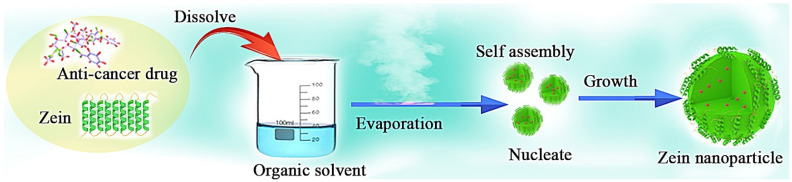
The formation process of anticancer drug zein-based NPs via EISA.

Evaporation-induced self-assembly (EISA) is a technique used to facilitate the self-assembly of nanostructures that involves the use of binary (or tertiary) solvents [33]. The change of the solution polarity drives the self-assembly of zein into various structures, such as nano- and micro-rods, spheres and thin films. When the drug is added to the solution, it can be easily encapsulated in the particles (Figure 4) [34]. For example, curcumin was loaded and embedded in zein NPs during solvent evaporation induction in the study of Cai et al. [35]. Wang et al. [36] developed an EISA method to prepare mixed zein phosphatidylcholine hybrid NPs to prevent triple-negative breast cancer. The results showed that the composite NPs had a high encapsulation rate (96.75 ± 1.41%). In vitro and in vivo experiments showed that the prepared DDSs had selectively high cytotoxicity against cancer cells and low cytotoxicity against normal cells.

#### 2.1.3. Anti-Solvent Self-Assembly

**Figure 5:**
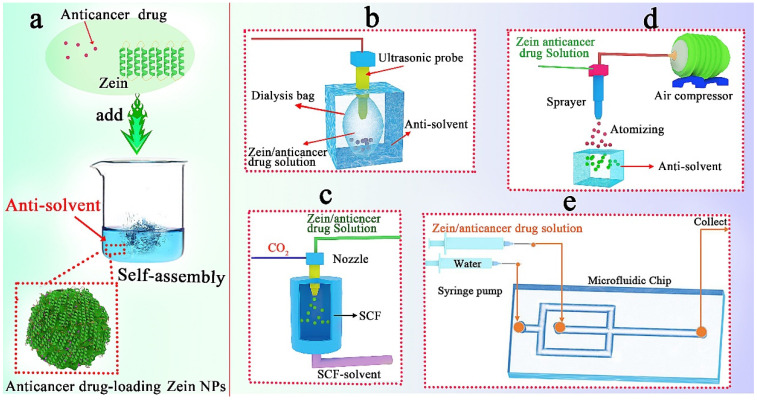
(**a**) The anti-solvent self-assembly process of zein, (**b**) the ultrasonic dialysis process, (**c**) the self-assembly process in a supercritical fluid, (**d**) atomizing/antisolvent precipitation process, (**e**) microfluidic technology.

Antisolvent precipitation (ASP), also known as the liquid–liquid dispersion or phase separation method, is commonly used to prepare anticancer drug-loading zein NPs [37]. Usually, deionized water is used as the anti-solvent and an aqueous ethanol solution is used as the solvent for zein. By adjusting the mixing rate and zein concentration, the properties of anticancer drug-loading zein NPs (such as size, dissolution rate, drug sustain-release, encapsulation efficiency and drug loading capacity) can be optimized to harvest the optimum cancer treatment effect. For example, zein–alginate oligosaccharide complex NPs were successfully prepared to encapsulate anticancer drug (curcumin) by the antisolvent precipitation method, where uniform and stable NPs were prepared at a 2:1 ratio of zein to alginate oligosaccharide [38]. As shown in Figure 5, there are some derivative methods of ASP.

(1) Ultrasonic dialysis process

The slow dialysis process is beneficial for the fine control of the change in solvent concentration; then, the size and morphology of zein-based NPs can be precisely controlled. In our preliminary work (Figure 5b), a built-in ultrasonic dialysis process (BUDP) was developed for the self-assembly of curcumin-loaded zein NPs with controllable particle size and narrow distribution [39]. Based on this method, Zhu et al. [29] prepared a zein–ZF-8 hybrid NP with pH-responsive drug delivery; Ye et al. [40] prepared pluronic-zein–curcumin DDSs, which showed an enhanced hydrophilicity and sustained-release behavior. It is worth noting that the high-energy ultrasonic probe was used to make a fast mixing of solution in BUDP. Therefore, a low temperature was needed to avoid the increase in crosslinking NPs.

(2) Self-assembly in supercritical fluid

Supercritical fluid (SCF) is any substance whose temperature and pressure are above the critical point [41]. SCF provides high levels of supersaturation, which is difficult in traditional technology [42]. As shown in Figure 5c, the antisolvent self-assembly in SCF is usually carried out by injecting the mixture solution of zein and drug into SC-CO_2_ in a high-pressure vessel through a coaxial nozzle. For example, a cancer-preventive substance (luteolin) was loaded into zein microparticles with high entrapment efficiencies (up to 82%) by an SCF process [43]. An SCF process can be combined with other methods to obtain complicated particles. Our preliminary work fabricated 10-hydroxycamptothecin (HCPT) nanocrystals by an SCF process. Then, we used these nanocrystals as templates or hydrophobic cores during the self-assembly process of zein molecules, resulting in the formation of HCPT nanocrystal-loaded zein microspheres [44]. Wang et al. [17] further modified this process to prepare an HCPT nano-drug delivery system, where folic acid and Au NPs were used to functionalize the DDS, leading to the target drug delivery and cell imaging.

(3) Atomizing/antisolvent precipitation process

By combining spray drying with antisolvent precipitation technologies, Wu et al. [45] established an atomizing/antisolvent precipitation (AAP, Figure 5d) method to prepare zein/soy lecithin/carboxymethyl chitosan core–shell NPs. The obtained NPs could change the crystal morphology of docetaxel, increase the dissolution rate of docetaxel, and exhibit sustained release behavior. Zhang et al. [46] further prepared zein-based NPs using this method for loading resveratrol. The NPs had a high zeta potential (−47.7 ± 0.66 mV) and a high encapsulation efficiency (91.32 ± 4.01%).

(4) Microfluidic technology

Recently, microfluidic technology has emerged as an effective means for the preparation of DDSs [47]. Different structure, constitution, morphology and size of the NPs can be obtained by control of microfluidic devices and chip geometries [48]. Figure 5e shows a preparation process of zein-based DDSs by microfluidic technology. In this process, a flow-focused microfluidics chip is used, and the self-assembly of zein is controlled by the flow rate, concentration and temperature of the zein solution. By using this millisecond solvent–antisolvent mixing technology, DDSs with high encapsulation efficiency (97.2 ± 0.6%) and loading capacity (11.1 ± 0.1%) were obtained [49]. Meewan et al. [50] prepared PEGylated zein-based NPs by a microfluidic method. The modified NPs showed enhanced stability and higher coumarin-6 uptake by melanoma cancer cells. Olenskyj et al. [51] fabricated zein NPs by microfluidics. It was found that increasing the total flow rate would result in small NPs and large PDI. In fact, microfluidics can be used not only for the synthesis of nano DDSs, but also for drug delivery and evaluation [52]. With the further development of microfluidics, it will become a potential force for clinically translatable nanotechnology in the future.

#### 2.1.4. Chemical Synthesis of Prodrugs

The bioavailability of many anticancer drugs is very low due to their poor drug solubility and decomposition in the gastrointestinal tract [32,53]. The prodrugs approach is a powerful strategy to improve the physicochemical, biopharmaceutical and pharmacokinetic properties of parent drug molecules [54]. Using this strategy, the drug can change its distribution, bioavailability and side effects. For example, a new zein-based prodrug was synthesized by covalent coupling of zein and PTX through disulfide bonds [24]. Animal experiments showed that the original size of the tumor significantly reduced after three doses.

### 2.2. Type of Pharmaceutical Preparation

Zein-based DDSs for cancer therapy have been designed as various pharmaceutical preparations, including NPs, micelles, hydrogels and films, as summarized in Table 1.

#### 2.2.1. Micelles

Zein is an amphipathic protein molecule that can be dissolved in an ethanol solution. The changes in the solvent polarity of solution lead to the rapid self-assembly process of zein, forming zein micelles with hydrophilic segments (shell) and hydrophobic segments (core). The hydrophobic core of zein micelles can load hydrophobic drugs with low solubility, while the hydrophilic shell can form a protective layer to protect drugs from harsh external environments and prolong the blood circulation time [63]. Therefore, zein-based micelles have shown great potential for cancer drug delivery. For example, Podaralla et al. [55] synthesized methoxy poly(ethylene glycol) (mPEG) conjugated zein biodegradable micelles, and curcumin was encapsulated in this DDS. The cell uptake of curcumin-loaded mPEG–zein micelles was higher than that of free curcumin in drug-resistant cancer cells; Gaber et al. [11] synthesized amphiphilic zein–chondroitin sulfate-based copolymeric micelles, which showed lower IC50 cytotoxicity against breast cancer cells.

#### 2.2.2. Hydrogel

Hydrogels have numerous advantages in drug delivery, including biocompatibility, low toxicity and good swelling behavior [64]. Gelation of zein can be formed through the cross-linking of hydrophilic chains in an aqueous microenvironment. The smart hydrogel DDSs can protect the drug from external stimuli such as heat, ionic strength, pH and electric field [65]. For example, Kaushik et al. [27] prepared doxorubicin (DOX)-encapsulated zein NPs by crosslinking pectin hydrogel. The obtained DDS exhibited a pH-responsive release in the acidic tumor microenvironment. Kunjiappan et al. [58] used a graft polymerization technique to prepare a zein–co-acrylic acid hydrogel. The formulation was optimized by a variety of zein, acrylic acid and initiator. Then, 5-fluorouracil and rutin were loaded in this optimized hydrogel. The results of cell experiments showed that these hydrogels could cause 50% death of breast cancer cells when the concentration was 52.5 μg/mL.

#### 2.2.3. Nanoparticles

**Figure 6:**
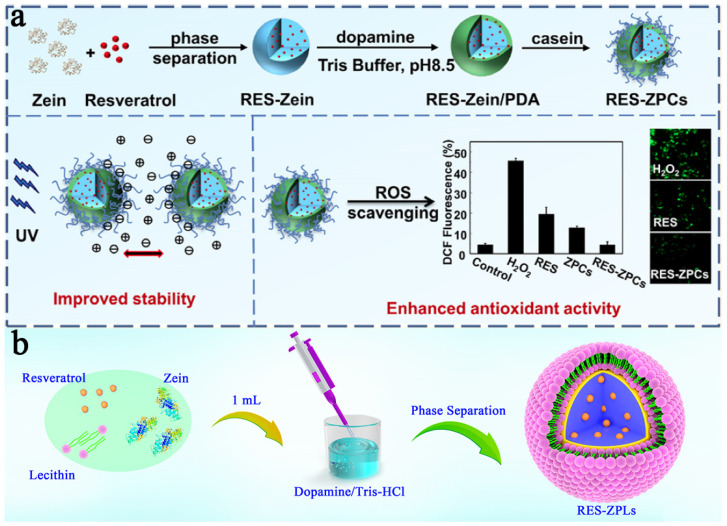
(**a**) The preparation process of RES–ZPCs, reproduced with permission from [67], American Chemical Society, 2018. (**b**) The preparation process of lecithin- and dopamine-modified zein NPs, reproduced with permission from [25], Springer Nature, 2019.

Zein NPs are easily prepared by a self-assembly process. However, the nanospherical zein with a small particle size (about 200 nm) possesses a higher specific surface area and tends to aggregate according to the principle of minimum system energy. Hence, stabilizers such as lecithin [59], casein [66] and dopamine [67] are used to enhance its stability. In our previous study [60], casein-coated zein-based core–shell NPs were prepared (Figure 6a). The results showed a highly active and stable characteristic, which enhanced approximately 5-fold and 2.5-fold antioxidant activity by DPPH and ABTS assays, respectively. Moreover, the lecithin and dopamine were used to fabricate a stable zein-based DDS (Figure 6b) [25]. The samples showed high stability under the conditions of a saline solution, various pH values, long-term storage and high-speed centrifugation.

Zein NPs possess many unique properties in drug delivery. Dong et al. [60] encapsulated DOX into zein NPs with a high encapsulation efficiency (90.06%) and loading efficiency (15.01 mg/g). The obtained zein NPs entered not only the cytoplasm, but also the cell nucleus. Moreover, zein NPs exhibited pH-responsive release and high cellular uptake behavior. Yu et al. [61] prepared maytansine-loaded zein NPs by phase separation, which exhibited a high tumor inhibition rate (97.3%) and high tumor accumulation ability in vitro and in vivo.

## 3. The Challenges and Solutions of Zein-Based Nanocarriers for Precise Cancer Therapy

Although zein shows many advantages in delivering various anticancer drugs, it has limitations in terms of burst release, non-specific targeting, concentration variations and side effects. To realize precise delivery of anticancer drugs, the modifications of zein-based nanocarriers should be explored.

### 3.1. Stable Zein-Based DDSs

The stability of DDSs is important for the delivery of antitumor drugs. Zein-based NPs, nano hydrogels and micelles possess small particle size and high surface energy, which tend to aggregate after short storage periods. Increasing zeta potential is a simple and effective method to improve their stability. For example, if the pH of the solution is changed from pH 7.0 to pH 4.0, the surface potential of the zein particle increases to 40 mV [68], which significantly increases the electrostatic repulsion. Therefore, the stability of zein-based DDSs is enhanced. To improve the stability of the zein solution, modification with, for example, lecithin [59], casein [66] or dopamine [67] is another good choice, but this increases the complexity of the preparation process. It is worth noting that the solution is not suitable for long-term storage and preparing zein-based DDS lyophilized powder is better. Therefore, it is necessary to explore the lyophilization process of zein. Unfortunately, it has not been well reported at present.

### 3.2. Targeting Zein-Based DDSs

Compared to conventional DDSs, targeting DDSs can reduce drug toxicity and resistance, enhance drug aggregation at the tumor site and avoid damage to healthy cells [69]. To ensure the successful delivery of anticancer drugs, targeting DDSs need to cross multiple biological barriers [70], avoid the elimination of immune systems [71] and reach and enter the targeted cancer cells quickly [72]. 

During the growth process of solid tumors, the vascular endothelium becomes more permeable than that in the healthy state [18], which is known as the EPR effect [73]. EPR effects depend on intrinsic tumor biological characteristics, nanocarrier size and cycle time [74]. PEGylation is commonly used by zein-based DDSs to enhance their EPR effects. Differing from passive targeting by EPR effects, active targeting DDSs are achieved by binding to tumor cell surface receptors or by simple adsorption [69]. The antitumor effect largely depends on the process of cell internalization, where the affinity between targeting ligands of DDSs and surface receptors of a tumor cell is an important criteria [16]. Table 2 lists the active targeting strategy of zein-based nanocarriers, i.e., conjugating with folic acid, transferrin, peptides and sugar residues.

#### 3.2.1. Conjugating with Folic Acid

**Figure 7:**
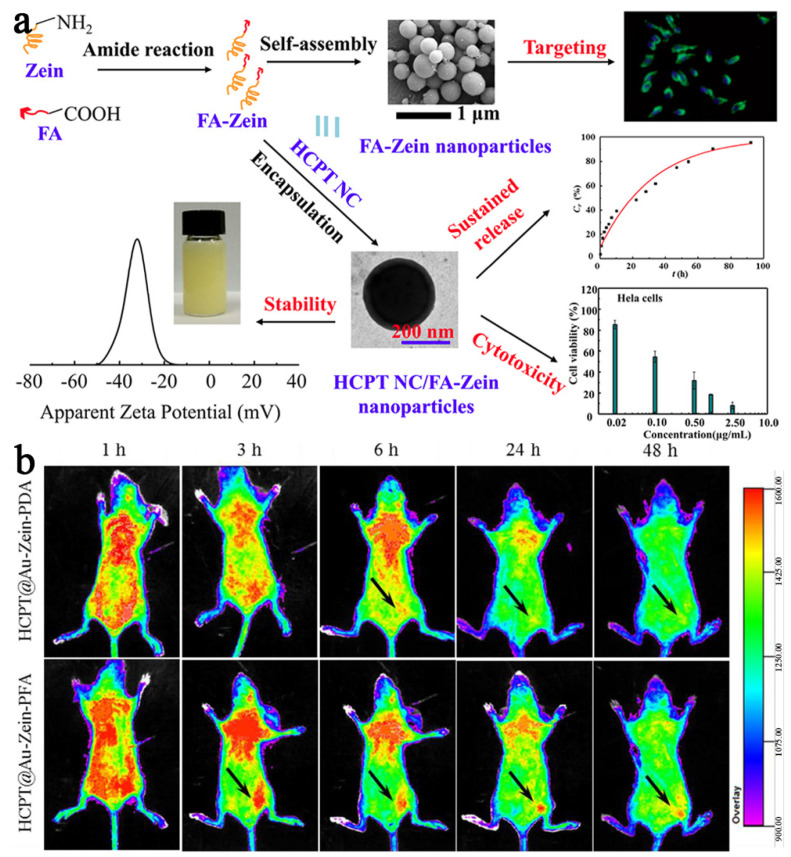
(**a**) The zein NP modified with FA to enhance its delivery performance, reproduced with permission from [75], American Chemical Society, 2017. (**b**) The biodistribution of zein NP modified with folate, reproduced with permission from [17], Elsevier, 2017.

The folate receptors’ expression in cancer cells is 500 times higher than that in healthy cells [79]. Therefore, nanocarriers are often modified with folic acid (FA) to enable effective targeted delivery of anticancer drugs. Liu et al. [75] modified the surface of zein NPs with FA for targeted delivery of HCPT (Figure 7a). The grafting degree increased from 2.26 ± 1.13 to 6.20 ± 0.28 (moL/moL) with the FA/zein ratio increasing from 10 to 85. Cytotoxicity studies showed that FA–zein could selectively target the delivery of the anticancer drug and reduce the non-specific toxicity to normal cells. Wang et al. [17] used AuNPs as the core and FA-conjugated amphiphilic zein–PDA as the shell to realize the targeted delivery of HCPT. The results (Figure 7b) showed that strong fluorescence was observed at the tumor site. Soe et al. [80] successfully prepared FA-modified zein NPs with small particle sizes (180 nm) and narrow PDI (0.22). The biological distribution of KB tumor-bearing mice was studied, and the hydrophobic drug PTX showed an enhanced ability of active targeted delivery. Wu et al. [45] prepared FA-conjugated zein-based NPs, which significantly improved the cytotoxicity of docetaxel to cancer cells in vitro.

#### 3.2.2. Grafting with Peptides

**Figure 8:**
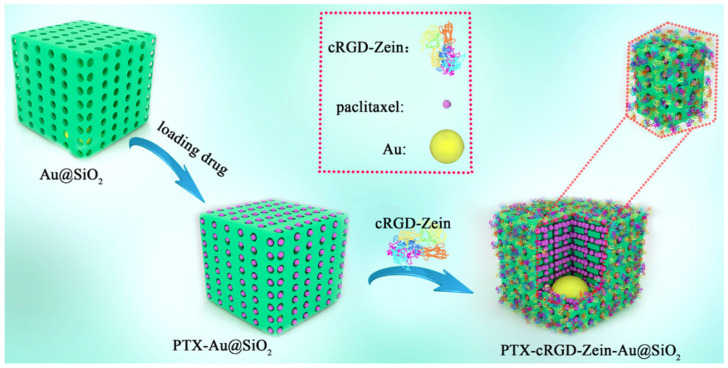
The preparation process of PTX–cRGD–Zein–Au@SiO_2_, reproduced with permission from [9], Elsevier, 2021.

Peptides, with low immunogenicity, non-toxic metabolites and enhanced retention in tumor sites, can bind to overexpressed receptors on the surface of cancer cells and can, therefore, be considered as promising active targeting ligands [81]. In our previous work [9], a bio-safe and targeted zein-based DDS was fabricated for chemo-photothermal therapy, where a cRGD peptide-grafted zein was successfully synthesized and coated on the surface of PTX-loaded Au@SiO_2_ NPs (Figure 8). The results of cell experiments showed that the obtained PTX–cRGD–Zein–Au@SiO_2_ NPs had strong antitumor activity and uptake ability of tumor cells. Similarly, Zhang et al. [77] prepared novel zein-based NPs for the delivery of dactolisib by using a brain-targeting peptide RVG29. The results showed that RVG29 could promote blood–brain tumor barrier penetration and enhance tumor cellular uptake.

#### 3.2.3. Modifying with Sugar Residues 

The cluster of differentiation 44 (CD44) is overexpressed in several types of cancers, including liver, ovarian, lung, brain and breast cancers. CD44 has a high affinity for sugar residues such as hyaluronic acid and chondroitin sulfate [82]. Zhang et al. [62] prepared core–shell NPs of zein and hyaluronic acid for the targeted delivery of honokiol. The results showed that adding hyaluronic acid could enhance intracellular uptake as well as cytotoxic and pro-apoptotic activities in vitro. The animal experiment indicated that the anti-tumor effect was improved due to the presence of CD44 receptor-mediated endocytosis. In another study, Li et al. [78] developed novel chondroitin sulfate hybrid zein-based NPs for targeted delivery of dactolisib. The obtained DDS could improve the colloidal stability and cellular uptake efficiency, which showed an effective accumulation of NPs in tumor tissues.

#### 3.2.4. Others

**Figure 9:**
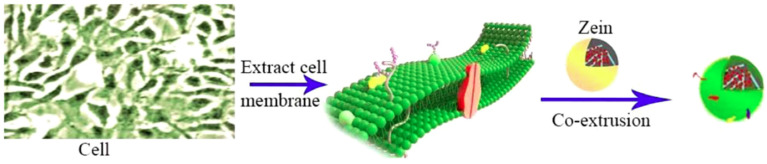
Schematic illustration of the preparation of CCM–PTX-α–zein cell membrane using co-extrusion technology, reproduced with permission from [7], Elsevier, 2023.

Cell membrane coating technology offers a new opportunity for precision drug delivery [15,83,84]. Using this technology, the highly complex cell membrane functionalities can be completely replicated. This endows the DDS with prolonged systemic circulation time, targeting capability and enhanced internalization [85]. In our previous work [7], the cancer cell membranes were coated on the surface of the zein NPs (Figure 9). The experiments of hemolysis and cell safety tests implied that the DDS was safe in the in vitro study. The cancer cell cytotoxicity, nuclear activity and cell uptake experiments showed that the DDS possessed enhanced homotype targeting ability.

Lactoferrin is a natural cationic glycoprotein, which is a member of the transferrin family. It can bind to transferrin receptors and lactoferrin membrane internalized receptors, which are overexpressed on the surface of cancer cells and blood–brain barrier cells [76]. Based on this unique character, transferrin is widely used to functionalize nanocarriers for active targeting delivery. Sabra et al. [56] successfully developed an amphiphilic polymeric micelle, which had a hydrophobic zein core and a hydrophilic lactoferrin shell. The hydrophilic lactoferrin shell could enhance the tumor targeting, reduce the interaction between micelles and serum proteins and prolong systemic circulation of the nanocarriers.

### 3.3. Responsive Zein-Based DDSs

The tumor microenvironment is the internal environment in the tumor growth process, which consists of cancer cells, blood vasculature, stromal cells, and extracellular matrix [86]. The tumor microenvironment is considered as the “soil” for tumor development and is an important part of tumor tissue. Compared with the normal internal environment of the human body, the tumor microenvironment has many unique biochemical characteristics, such as weak acidity, strong reduction, strong oxidation, low oxygen and up-regulation of enzyme secretion [87,88]. Based on the understanding of the unique characteristics of the tumor microenvironment, stimulating responsive cancer therapies have been developed to realize triggering drug release [89]. Nowadays, zein-based nanocarriers have been designed to achieve a rapid response to various stimuli, including pH, redox, temperature and photo- and magnetic fields [90].

#### 3.3.1. pH Response

Due to uncontrolled tumor growth and abnormal gene expression, the physiological characteristics of tumor tissues are exhibited significantly differently from those of normal tissues [91]. Irregular angiogenesis at rapidly growing tumor sites leads to the deficiency of nutrients and oxygen; thus, glycolytic metabolism occurs, and acidic metabolites are generated in the tumor interstitium [92]. It has been reported that the approximate pH range of the tumor site is from 6.5 to 7.0, and pH in normal tissue is about 7.4 [93]. Liang et al. [94] developed a metal–polyphenol-coated zein DDS to deliver DOX to tumor tissues. Cleavage of the metal–polyphenol network was easily disrupted in lower pH, resulting in pH-responsive drug delivery. Anirudhan et al. [95] prepared pH-responsive nanocarriers using aminated mesoporous silica NPs as a backbone and glycyrrhetinic acid-conjugated zein as a shell. The release of 5-fluorouracil and curcumin was higher at pH 5.5 than that at pH 7.4. Kaushik et al. [27] reported a pH-responsive gel of zein-based nanocarriers. The controlled release of DOX was achieved at pH 6.4 at 37 °C. The prepared hydrogel could facilitate the pH-responsive release of DOX into the cytoplasmic acidic environment of HeLa cells.

#### 3.3.2. Redox Response

It is well known that the redox potential between healthy and tumor cells is different, which provides a strategy for developing stimulus-responsive DDSs. Glutathione (GSH) is an important intracellular antioxidant that regulates the redox state of cells [96]. Disulfide bonds are typical redox-reactive covalent bonds. GSH can control the cytoreductive environment by using excessive reactive oxygen species (ROS) to form and cleave disulfide bonds [97]. It has been reported that the concentration of GSH in tumor cells is significantly higher than that in normal cells [98,99]. Hence, redox-responsive DDSs offer an opportunity for precise cancer therapy. For example, Hou et al. [24] synthesized a novel redox-triggered zein-based DDS. In vitro release assays showed that PTX was not released without GSH. The antitumor efficiency in vitro and in vivo showed that redox-triggered DDSs tended to approach cancer cells more than normal cells.

#### 3.3.3. Photo Response

Photodynamic therapy (PDT) is a novel medical tool that uses photosensitizers to kill cancer cells [100]. The light-sensitive material can absorb light energy and convert it into heat energy, which can promote anticancer drug release and inhibit tumor cell activity. The photo response systems are non-invasive, which can increase the therapeutic effect and patient compliance [101]. Indocyanine green (ICG), a near-infrared dye, has potential application in the cancer phototherapy photosensitizer. After exposure to near infrared ray (NIR), ICG can generate ROS and cause tumor cell death. Li et al. [102] reported ICG-encapsulated zein–phosphatidylcholine NPs for the phototherapy of cancer. Cancer cell experiment analysis showed that encapsulated ICG enhanced the photocytotoxicity greatly. Yu et al. [14] have fabricated docetaxel-loaded zein NPs coated by a green tea polyphenols/iron coordination complex (GTP/Fe^III^, a photothermal agent) to achieve a combined chemical–immune–photothermal therapy. The photothermal conversion efficiency of NPs was 37.82% under 808 nm NIR laser irradiation (2 W/cm^2^). In vitro cytotoxicity studies and in vivo antitumor experiments showed that the NPs exhibited a strong inhibitory effect in tumor cells.

#### 3.3.4. Magnetic Response

**Figure 10:**
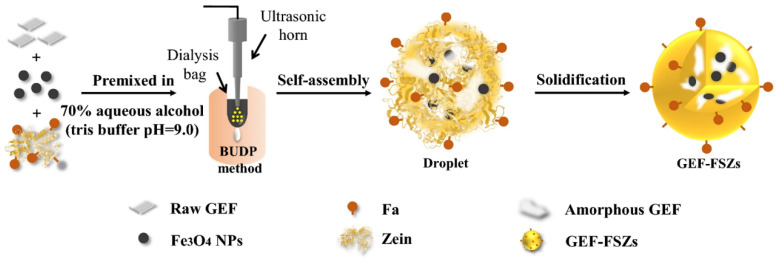
The preparation process of a magnetic zein-based nanocomplex, reproduced with permission from [105], Springer Nature, 2018.

Magnetic NPs for biomedical applications depend not only on their intrinsic magnetic properties, but also on their biophysical properties under physiological conditions [103]. Magnetic NPs are usually divided into pure metals, metal oxides and magnetic nanocomposites. Magnetically responsive NPs can accumulate at the tumor site under precise spatial guidance of in vitro magnetic field [104]. Embedding the magnetic NPs into zein-based DDSs enhances the drug delivery performance. Pang et al. [105] fabricated a magnetic zein-based nanocomplex by adding the Fe_3_O_4_ into zein NPs during the self-assembly process (Figure 10). The data of in vitro cellular uptake analysis showed that the fluorescence signal was over 95.6% for cells within 10 min under control of an external magnetic field, suggesting that a magnetic field could enhance the cellular uptake in vitro.

### 3.4. Hybrid Zein-Based DDSs

For precise cancer therapy, zein-based nanocarriers exhibit some shortcomings such as stability, targeting and hydrophobicity [106]. To enhance the performance of zein NPs and optimize their therapeutic effects, it is necessary to design rational nanocarriers. Fabricating hybrid zein-based DDSs can achieve different anticancer effects by combining the advantages of each component, such as zein–polysaccharide hybrid NPs, zein–protein hybrid NPs, zein–surfactant hybrid NPs and zein–inorganic hybrid NPs. The zein-based hybrid NPs can be prepared by the driving forces of electrostatic, hydrophobic and hydrogen bonding interaction forces [107]. Under this interaction, co-assembly between the zein and hybrid NPs occurs; integrating their advantages into one unit can obtain precise cancer therapy.

#### 3.4.1. Zein–Polysaccharide Hybrid NPs

Polysaccharide-based NPs have promising potential in biomedical applications [108]. Polysaccharides are widely used for drug delivery in cancer therapy because of their advantages, including wide source, low cost, good biological activity and biodegradability [109]. In addition, polysaccharides have a lot of reactive functional groups, such as amino, hydroxyl and carboxyl moieties, which are beneficial for drug conjugation and physicochemical crosslinking with other proteins. Liu et al. [110] reported a zein formulation with three polysaccharides. In vitro and in vivo studies demonstrated that the addition of polysaccharides could enhance the delivery and accumulation of curcumin in colorectal cancer tumors. Ye et al. [111] prepared alginate-stabilized zein NPs for DOX delivery by flash nanoprecipitation. The addition of alginate could reduce the burst release of DOX and prolong the action time of drugs, thus enhancing the antitumor effect. Li et al. [78] prepared a polysaccharide (chondroitin sulfate) and zein hybrid NPs for delivery of docetaxel. In vitro and in vivo studies showed that chondroitin sulfate could improve the colloidal stability and cellular uptake efficiency of zein NPs. In addition, zein/chondroitin sulfate NPs could accumulate in tumor tissues without significant systemic toxicity.

#### 3.4.2. Zein–Protein Hybrid NPs

Hybrid with hydrophilic proteins is an effective method to improve the performance of zein-based NPs. Lu et al. [112] used hydrophilic animal protein (silk fibroin) and zein to prepare hybrid NPs. In this study, PTX and curcumin were encapsulated in hybrid NPs, and the inhibition ability of tumor cell growth was shown to be stronger than that of free PTX or curcumin. Chen et al. [12] prepared hybrid NPs with an optimal weight ratio of zein/bovine serum albumin (BSA) of 1:2. Among them, curcumin had higher binding strength to zein/BSA NPs, and the NPs exhibited high encapsulation efficiency and storage stability. Similarly, lipid can also be used to improve the performance of zein-based NPs. Kamel et al. [113] designed novel hybrid lipid nanocore–protein shell NPs (HLPNPs) using lipid as a core and zein as a shell. All-trans retinoic acid and genistein were loaded into the NPs. In vivo anticancer efficacy of HLPNPs showed the lowest lung lesion numbers, along with significant decrease in metastatic foci diameters and tumor biomarkers.

#### 3.4.3. Zein–Surfactant Hybrid NPs

In the development process of drug formulations, surfactants are often used as wetting agents to improve the dissolution of insoluble drugs [114]. In order to screen stabilizers for zein-based nanocarriers, Gagliardi et al. [115] applied Tween 80, Poloxam 188 and sodium deoxycholate to prepare zein–surfactant hybrid NPs. The physicochemical characterizations of the NPs were evaluated to optimize the drug formulation. Among them, sodium deoxycholate-based zein NPs exhibited a small size (~100 nm), low DPI and negative surface charge (−30 mV). After that, Gagliardi et al. [116] loaded the PTX into the fabricated sodium deoxycholate-stable zein NPs. The obtained DDSs could effectively retain PTX and increase toxicity in different cancer cell lines. Ye et al. [40] prepared pluronic–zein–curcumin DDSs, which improved the performance of zein in hydrophilicity, stability and drug delivery. After being modified with pluronic, the surface contact angle was reduced by 33.1–62.8% and DDS remained stable under pH and salinity conditions.

#### 3.4.4. Zein–Inorganic Hybrid NPs

Inorganic NPs (such as gold, silicon, carbon nanotubes and iron oxide) have large surface area, controllable structure and special optical properties, which are commonly used to enhance the performance of DDSs [117]. Zein–inorganic NPs have been intensively investigated to combine the advantages of zein and inorganic nanocarriers [118]. Chauhan et al. [8] prepared gold-deposited zein nanoshells (AuZNS) by depositing thin layers of gold on ethylene glycol chitosan-stabilized zein NPs. The AuZNS with a particle size of 100 nm could realize photothermal cancer therapy, and the cell viability of two different cancer cell lines (MCF-7 and C33A) was reduced to 25% under 808 nm laser irradiation for 5 min. Anirudhan et al. [95] used zein as a shell to encapsulate mesoporous silica for preventing premature drug leakage and realizing sustained release. Bahmani et al. [119] used zein-based multi-walled carbon nanotubes (MWCNTs) to prepared nanofibers, and DOX and PTX were loaded. These composite core–shell nanofibers showed the synergistic therapeutic effects of MWCNTs and anticancer drugs, which had a high biocompatibility, with 84% of MCF-7 cancer cells killed at a low dosage.

## 4. Outlook

Although zein-based DDSs have exhibited a powerful performance for cancer therapy, there are still some challenges for their clinical translation. (1) Similarly with other nano-DDSs, the stability of zein-based DDSs is the key point for their clinical translation. NPs with a small size have a high surface energy, which results in the aggregation of samples. Surface modification is a good strategy for improving stability, such as modification with lecithin, casein and dopamine. (2) The hydrophobicity of zein will be easily cleared by immune cells. Although PEGylation and cell membrane technology have developed, there is still a long way to go for their clinical application. (3) Challenges also remain in the large-scale production of zein-based DDSs with uniform particle size, high encapsulation rate and drug loading capacity. This is a need to develop new techniques and instruments to further optimize preparation methods. Additionally, how to reduce batch-to-batch errors is a big puzzle. Recently, microfluidics technology has shown promising potential for solving this challenge, but industrial-grade equipment and technical theory should be further developed. Therefore, there are current challenges but also promising opportunities for zein-based nanocarriers in clinical translation.

## 5. Conclusions

Cancer has undoubtedly become one of the most important topics for public health problems. Although tremendous efforts have been paid in the past decade, precise anticancer drug delivery is underway and has yet to be fully realized. Recently, zein-based nanocarriers have emerged as a new generation of anticancer DDSs due to their specific promising physicochemical characteristics. The emerging advanced technology for preparing zein-based DDS has played an important role in the preparation of stable, targeting delivery, stimuli-responsive and multifunctional-hybrid zein-based NPs. In this review, the recent progress in zein-based nanocarriers for precise anticancer drug delivery is summarized. Novel methods by using ultrasonic dialysis, SCF and microfluidic technology have been extensively developed toward the control of drug loading. Zein-based NPs, micelles, hydrogels and films have been prepared for cancer therapy. Additionally, to realize precise delivery of anticancer drugs, stable, targeting, responsive and hybrid zein-based DDSs have been developed. However, most of them are still at an early stage, and there are many challenges for their clinical translation. Potential efforts must be made to the specific modifications of zein-based carriers, including controllable preparation, large-scale production and clinical study. With continued innovation and development, the clinical translation of zein-based nanocarriers for precise cancer therapy will finally come true.

## Figures and Tables

**Table 1:** Examples of various zein-based DDSs for cancer therapy.

Carrier	Drug	Loading Strategy	Formulation	Characterization	Cancer Cell	Administration Route	Reference
hyaluronic acid–zein	curcumin	PCL	Nanogels	in vitro/in vivo	CT26, NIH3T3	injection	[31]
Zein–Lipoid-S75	exemestane and resveratrol	PCL	NPs	in vitro/in vivo	MCF-7, 4T1	oral gavage	[32]
Phosphatidylcholine–zein	isoliquiritigenin	EISA	NPs	in vitro/in vivo	4T1, MIHA	oral gavage	[36]
Pluronic–zein	curcumin	ASP	NPs	in vitro	A549	-	[40]
AuNPS–zein	HCPT	ASP	NPs	in vitro/in vivo	KB, Hela, A549	injection	[17]
Folate–zein	docetaxel	ASP	NPs	in vitro	MCF-7, SKOV-3	-	[45]
PEG–zein	coumarin-6	ASP	NPs	in vitro	B16-F10-luc-G5	-	[50]
mPEG–zein	curcumin	ASP	Micelles	in vitro/in vivo	NCI/ADR-RES	injection	[55]
chondroitin sulfate–zein	etoposide	ASP	Micelles	in vitro/in vivo	MCF-7	injection	[11]
Zein–lactoferrin	rapamycin and wogonin	ASP	Micelles	in vitro/in vivo	MCF-7	injection	[56]
mPEG–zein	curcumin	ASP	Micelles	in vitro	HepG2	-	[57]
Pectin–zein	DOX	PCL	Hydrogels	in vitro	Cervical cells	-	[27]
Zein–co-acrylic acid	5-fluorouracil and rutin	PCL	Hydrogels	in vitro	MDA-MB-231, MCF-7	-	[58]
Zein–lecithin	carvacrol	ASP	NPs	in vitro	SW480	-	[59]
zein	DOX	ASP	NPs	in vitro	HeLa	-	[60]
zein	maytansine	ASP	NPs	in vitro	A549	-	[61]
zein/hyaluronic acid	honokiol	ASP	NPs	in vitro/in vivo	4T1	injection	[62]

**Table 2:** Active targeting strategy of zein-based nanocarriers.

Class	Ligand	Drug	Target	Reference
Folic acid	Folic acid	HCPT	Folate receptor	[75]
Transferrin	Lactoferrin	Rapamycin and wolfsbane	Lactoferrin receptor	[76]
Peptides Peptides	cRGD	PTX	Integrin	[9]
	RVG29	Dactolisib	Nicotinic acetylcholine receptor	[77]
Sugar residues Sugar residues	Hyaluronic acid	Honokiol	CD44 receptor	[62]
	Chondroitin sulfate	DOX	CD44 receptor	[78]

## Data Availability

Not applicable.
